# Supplementary material for: Enhancement of Growth and Secondary Metabolites by the Combined Treatment of Trace Elements and Hydrogen Water in Wheat Sprouts
Source: Int J Mol Sci. 2023 Nov 25;24(23):16742. doi: 10.3390/ijms242316742 (PMC10706805; doi:10.3390/ijms242316742)
Supplement: Supplementary file 1 [file ijms-24-16742-s001.zip › ijms-2708581-supplementary.pdf]

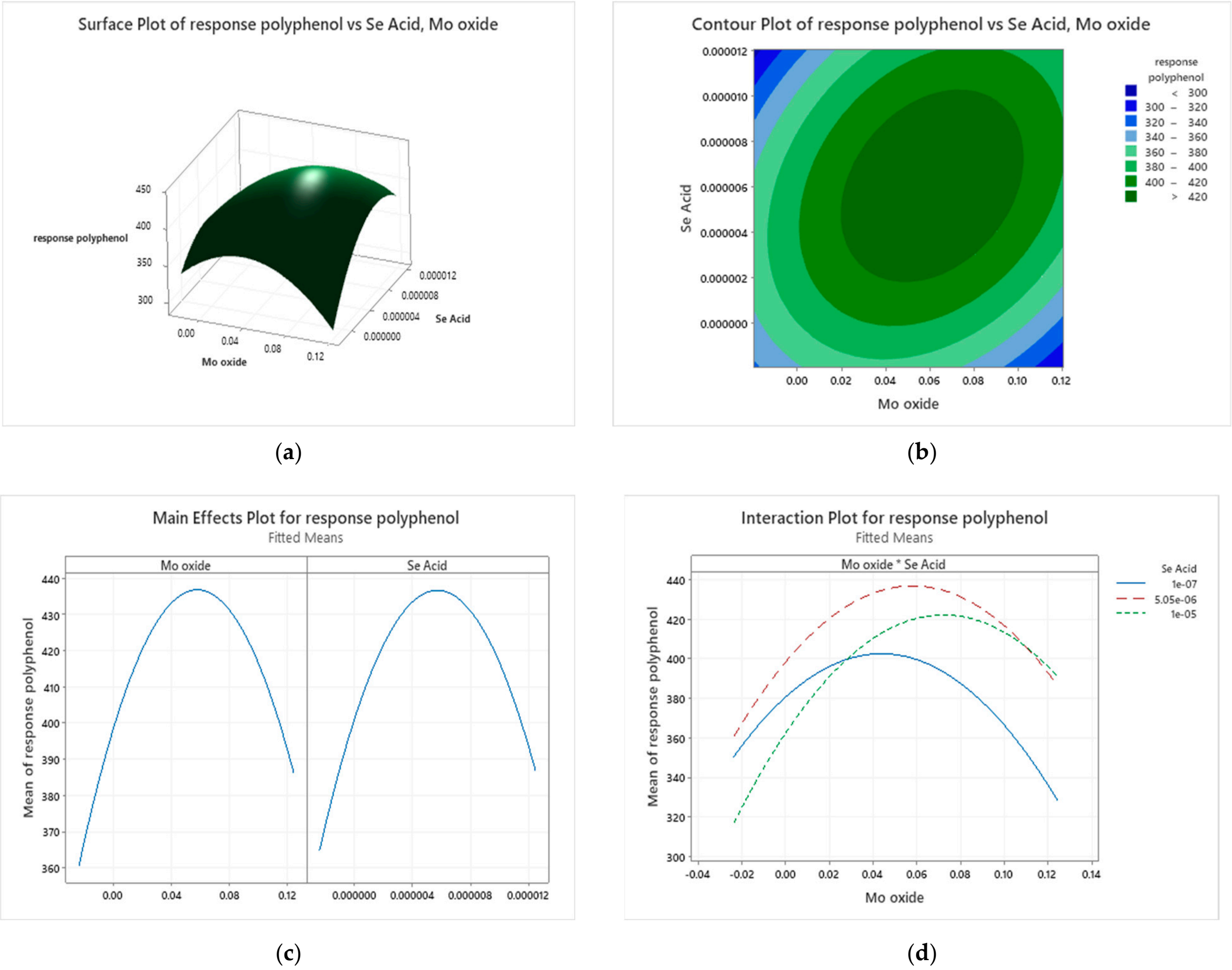

Figure S1. Effects of Mo oxide and Se acid treated with HW (**a**) surface plot, (**b**) counter plot, (**c**) factorial plot and (**d**) interaction plot of the total polyphenol content (mg/mL). The blue, green, and red lines represent the interaction at different concentrations of Se acid and Mo oxide. The factorial plot, meanwhile, depicts the optimal concentration ranges of each treatment and interaction plot indicating the significant interaction between trace elements.

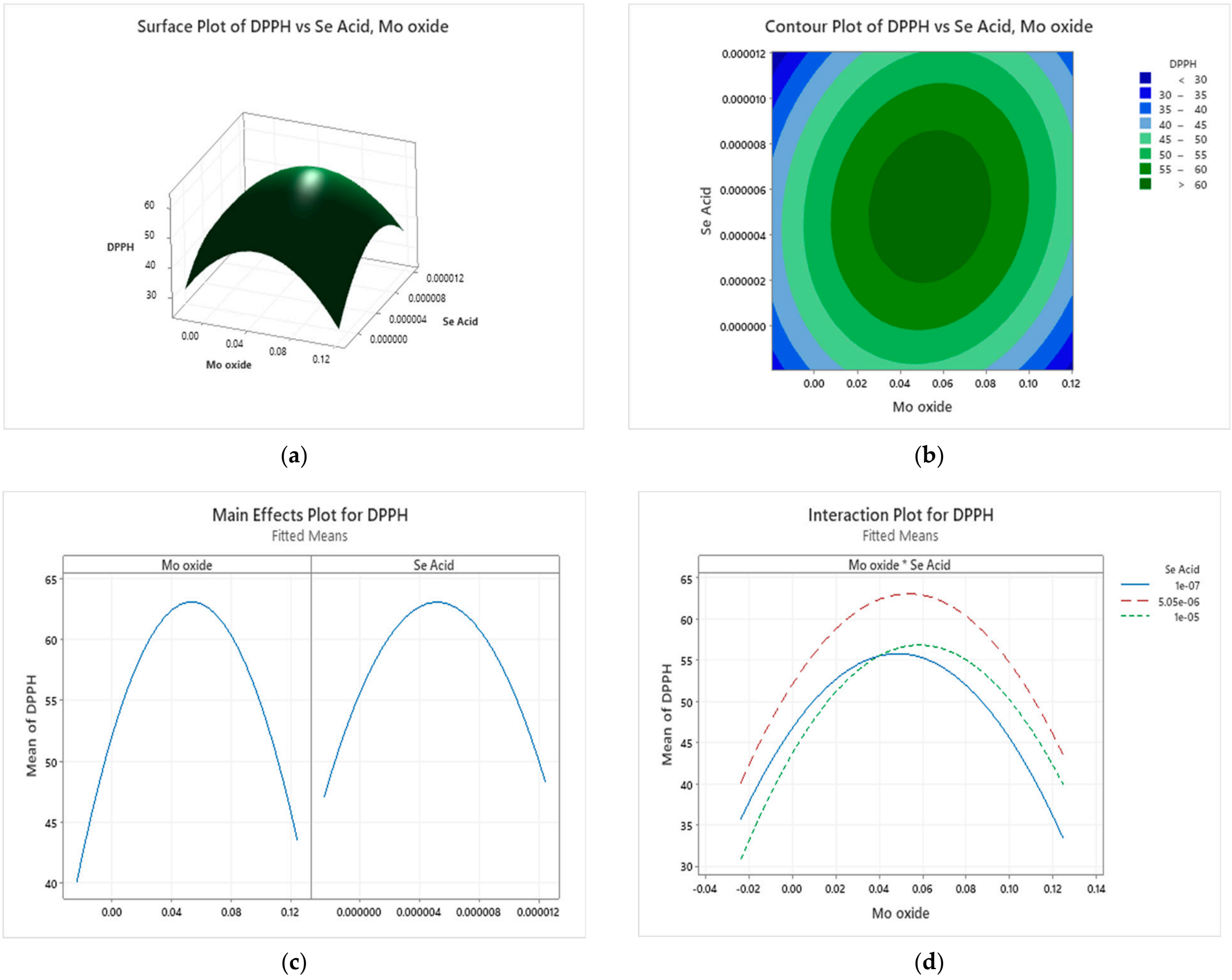

Figure S2. Effects of Mo oxide and Se acid treated with HW (**a**) surface plot, (**b**) counter plot, (**c**) factorial plot and (**d**) interaction plot of the antioxidant capacity (DPPH) (mg/mL). The blue, green, and red lines represent the interaction at different concentrations of Se acid and Mo oxide. The factorial plot, meanwhile, depicts the optimal concentration ranges of each treatment and interaction plot indicating the significant interaction between trace elements.

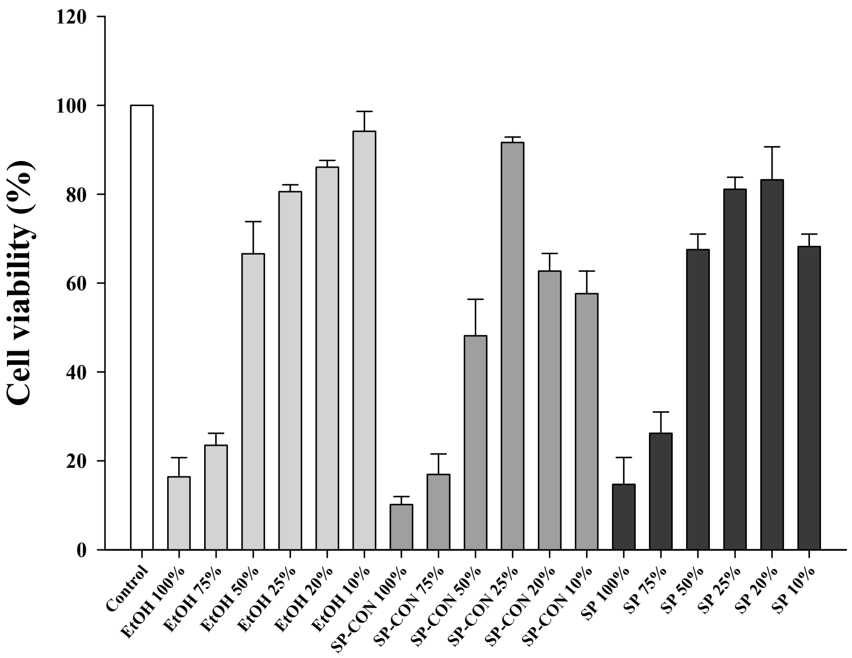

Figure S3. Cell viability assessment using the MTT assay; the cells were treated with different concentrations.
